# Biocompatible and Thermoresistant Hydrogels Based on Collagen and Chitosan

**DOI:** 10.3390/polym14020272

**Published:** 2022-01-10

**Authors:** Pablo Sánchez-Cid, Mercedes Jiménez-Rosado, José Fernando Rubio-Valle, Alberto Romero, Francisco J. Ostos, Mohammed Rafii-El-Idrissi Benhnia, Victor Perez-Puyana

**Affiliations:** 1 Chemical Engineering Department, Faculty of Chemistry, University of Seville, 41012 Seville, Spain; pscb14495@yahoo.es (P.S.-C.); alromero@us.es (A.R.); 2 Pro2TecS-Chemical Product and Process Technology Research Centre, Chemical Engineering Department, University of Huelva, 21071 Huelva, Spain; josefernando.rubio@diq.uhu.es; 3 Clinical Unit of Infectious Diseases, Microbiology and Preventive Medicine, Institute of Biomedicine of Seville (IBiS), Virgen del Rocío University Hospital, CSIC, University of Seville, 41013 Seville, Spain; fostos@us.es (F.J.O.); rafiim@us.es (M.R.-E.-I.B.); 4 Department of Medical Biochemistry, Molecular Biology, and Immunology, School of Medicine, University of Seville, 41009 Seville, Spain

**Keywords:** collagen, chitosan, hydrogels, mechanical properties, microscopy, cell viability

## Abstract

Hydrogels are considered good biomaterials for soft tissue regeneration. In this sense, collagen is the most used raw material to develop hydrogels, due to its high biocompatibility. However, its low mechanical resistance, thermal stability and pH instability have generated the need to look for alternatives to its use. In this sense, the combination of collagen with another raw material (i.e., polysaccharides) can improve the final properties of hydrogels. For this reason, the main objective of this work was the development of hydrogels based on collagen and chitosan. The mechanical, thermal and microstructural properties of the hydrogels formed with different ratios of collagen/chitosan (100/0, 75/25, 50/50, 25/75 and 0/100) were evaluated after being processed by two variants of a protocol consisting in two stages: a pH change towards pH 7 and a temperature drop towards 4 °C. The main results showed that depending on the protocol, the physicochemical and microstructural properties of the hybrid hydrogels were similar to the unitary system depending on the stage carried out in first place, obtaining FTIR peaks with similar intensity or a more porous structure when chitosan was first gelled, instead of collagen. As a conclusion, the synergy between collagen and chitosan improved the properties of the hydrogels, showing good thermomechanical properties and cell viability to be used as potential biomaterials for Tissue Engineering.

## 1. Introduction

Hydrogels are polymeric networks capable of absorbing up to thousands of times their dry weight of water without dissolving or losing their structural integrity [1]. This water absorption produces a swelling of the hydrogel, reaching its limit when the osmotic and cohesive forces are in equilibrium [2]. Hydrogels can be physical or chemical depending on the crosslinking carried out. In this way, physical hydrogels have non-permanent physical bonds, such as Van der Waals interactions or hydrogen bonds, while chemical hydrogels are composed of covalent bonds, which are stronger [3]. For this reason, physical hydrogels are less stable, being dependent on external stimuli such as ionic strength, solvents, pH or temperature; while chemical ones tend to have greater mechanical resistance, being less dependent on external stimuli.

The technique used to process these hydrogels depends on the interactions [4]. Physical hydrogels can be manufactured by cooling the polymer solution, ionic interactions, complex coacervation, hydrogen bonding, maturation or freezing. On the other hand, chemical hydrogels are produced by incorporating chemical or enzymatic crosslinking agents, grafting or radiation. Most of the hydrogels studied to date are physical, with cooling being used as the preferred processing technique. Other authors made poloxamer-heparin/gellan gum and gelatin hydrogels, respectively, though this processing technique [5,6]. Furthermore, Perez-Puyana et al. studied the influence of the cooling parameters (i.e., temperature and time) on the collagen-based hydrogel properties [7].

Regarding applications, hydrogels are used mainly in the biomedical field [1]. Thus, they were used as controlled drug release systems [8,9], wound dressing [10], hygiene products [11], contact lenses [12] and tissue engineering [13]. In the latter field, hydrogels can be used as scaffolds, whose functions are to act as extracellular matrix, drug delivery systems and antimicrobial agents during the recovery of the tissue and to support the cells and growth factors that favor the recovery of the tissue [14]. Their wide use in biomedical applications is due to the fact that hydrogels have a high biocompatibility. In addition, they have a soft touch and are flexible, which causes less irritation in the implantation area, and they are highly permeable, facilitating the exchange of oxygen, nutrients and metabolites in the cells [15]. Finally, they have a porous structure where cells can be accommodated, being able to easily penetrate and proliferate [1].

Hydrogels used in tissue engineering are usually composed of biopolymers due to their structure and properties, such as low antigenicity and inflammation, high affinity to water, adequate cytotoxic responses, biocompatibility, biodegradability and mucoadhesiveness [16]. Among them, collagen is the most studied, since it is highly biocompatible and it is the most abundant protein in animals (it forms the extracellular matrix) [17]. However, collagen hydrogels are usually obtained by cooling, giving them low mechanical properties, poor thermal stability and rapid degradation, limiting their use. Against this, crosslinking agents have been used to improve the properties of hydrogels, although most of them have limitations, above all of cytotoxicity [18]. For this reason, the use of biocomposites is being recently evaluated, where the high biocompatibility of collagen is mixed with another material that improves its mechanical properties [19]. Thus, Kanungo et al. [20] and Coombes et al. [21] used polycaprolactone (PCL) to improve the mechanical properties of collagen materials and Lv et al. [22] evaluated the inclusion of fibroin to improve the thermal stability of collagen hydrogels.

Among the materials proposed in the literature, the combination with polysaccharides is a potential alternative that can present synergies that favor the properties of scaffolds [23]. Chitosan stands out among them due to its high biocompatibility, biodegradability, low toxicity, cytocompatibility, mucoadhesiveness, anti-inflammatory activity, antibacterial and antifungal activity and wound healing activity [24,25]. In addition, chitosan allows the generation of covalent bonds in the hydrogel, which improves its mechanical properties and stability [26,27,28]. When chitosan is combined with collagen electrostatic interaction are formed between the carbonyl groups of collagen and the amino groups of chitosan. Furthermore, the rest of the amino carboxylic and ammonium groups that do not dissociate form H-bonding interaction. These physical interactions between both biopolymers are the reason why there is a great interest in their joint use in the formation of hydrogels [29]. In this way, Tan et al. saw the great potential of the joint use of collagen and chitosan and studied their interconnection in hydrogels [30]. In addition, this combination was used by Deng et al. to develop a hybrid collagen–chitosan hydrogel by chemical crosslinked to improve the properties of collagen hydrogel in cardiovascular tissue regeneration [31]; by McBane et al. in islet transplantation [32]; and by Gilarska et al. in bone tissue remodeling [33]. Nevertheless, all the combinations of collagen and chitosan hydrogels studied so far require a chemical crosslinking to generate the structure, which complicates its obtaining and can compromise their cytotoxicity and cell viability. In this way, the use of physical crosslinking to obtain a hybrid collagen/chitosan hydrogel could be a breakthrough in this sector.

In this context, the main objective of this work was the development of hybrid collagen/chitosan hydrogels by physical crosslinking. According to previous studies, chitosan forms a gel when there is a change in the solution towards pH 7 [27], due to intermolecular interactions of a hydrophobic nature are favored with this pH change [34]. On the other hand, pork type I collagen showed a cold thermo-setting behavior. In other words, a treatment at low temperature may induce collagen gelation, thanks to the Van der Waals interactions and hydrogen bonds that promote long, aligned and static fiber structure [35,36]. In this sense, authors hypothesized that combining both effects could obtain suitable hybrid hydrogels from collagen and chitosan though a physical crosslinking. For this reason, a protocol was established with two stages: (I) a pH change (to pH 7) and (II) a temperature drop (to 4 °C). Nevertheless, the order of succession of these key steps could generate important changes in the obtained hydrogels. So, two different protocols were developed varying the order of the stages: (I)–(II) and (II)-(I) to evaluate if there are significant differences in the produced hydrogels with the change of the order of gelation of the biopolymers. In addition, different ratios of collagen/chitosan (100/0, 75/25, 50/50, 25/75 and 0/100) were used to evaluate the synergic effect of their combination. The expected outcome of the study was the improved properties of the hybrid systems based on collagen and chitosan compared to the unitary systems, in terms of their thermorheological, microstructural and biological properties.

## 2. Materials and Methods

### 2.1. Materials

Hydrogels were formed by the combination of two biopolymers: collagen (CG) and chitosan (CH). For this, pork type I collagen (>90 wt% protein, denaturation degree of 75% and M_W_ = 30,000 g/mol) supplied by Protein Solutions (Essentia Protein Solutions, Grasten, Denmark), and low molecular weight chitosan (M_W_ = 130,000 g/mol and deacetylation degree 75–85%) provided by Sigma Aldrich S.A. (Taufkirchen, Germany) were used as raw materials. In addition, acetic acid (0.05 M and pH 3.2), supplied by Panreac Química S.A. (Barcelona, Spain), was used as solvent due to the complete solubility of CG and CH in it [23].

### 2.2. Processing of Hydrogels

**Figure 1:**
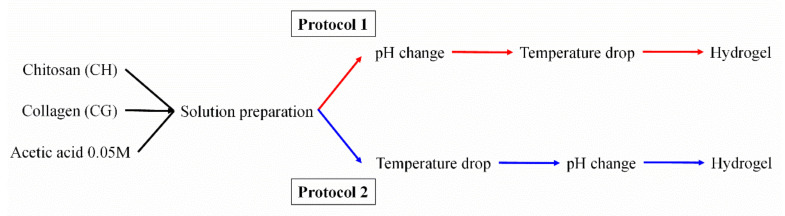
Scheme of the different protocols followed.

Hydrogels were processed with a three-stage process, using two different protocols (Figure 1). In both cases, the solutions were firstly prepared at a concentration of 15 mg biopolymer/mL solvent (1.5 wt.% of total biopolymer concentration). Different ratios of CG/CH were used: 100/0, 75/25, 50/50/, 25/75 and 0/100. The solutions were magnetically stirred at 300 rpm and 50 °C for 1 h. In the next step, the differentiation between the two protocols began. In protocol 1 (P1), a pH change from 3.2 to 7.0 was first carried out by adding 6 M NaOH sprayed, and subsequently it was placed in a fridge to drop the temperature to 4 °C for 24 h. In contrast, these two steps were performed in reverse in protocol 2 (P2).

It is worth mentioning that both stages are necessary, since pH change causes gelling of CH, while the temperature drop helps the gelation of CG (data not shown). Thus, the change in the sequence of these steps causes a first gelation of CH (protocol 1) or CG (protocol 2).

### 2.3. Rheological Characterization

Rheological characterization was carried out to evaluate the physical and mechanical properties of the hydrogels. Different tests were performed in a controlled-stress rheometer AR2000 (TA Instruments, New Castle, DE, USA) with plate-to-plate serrated aluminum geometry (diameter: 40 mm).

- Strain sweep tests: these trials were performed to evaluate the linear viscoelastic region, in which stress and strain have a linear relationship without altering the structure of the sample, and the critical strain (last strain in the linear viscoelastic region). These tests were carried out at between 0.1 and 100% of strain and at a constant frequency of 1 Hz and 4 °C.
- Frequency sweep tests: These analyses were performed at between 0.02 and 20 Hz and at a constant strain of 2% (below of the critical strain) and 4 °C. Thus, the behavior of the elastic (G′) and viscous (G″) moduli was evaluated in the entire frequency range studied. In addition, G′ and loss tangent (tan δ = G″/G′) at 1 Hz (G′_1_ and tan δ_1_, respectively) were evaluated to improve the comparison between the systems.
- Flow curves: These tests were carried out at between 0.02 and 200 s^−1^ of shear rate (γ) and at a constant strain of 2% and 4 °C. In this way, these tests allow the systems to be categorized as Newtonian, dilatant or pseudoplastic according to the dependence of viscosity on shear rate.
- Temperature ramp tests: The stability of hydrogels with temperature is an important aspect of these systems since they will be incorporated in humans. Thus, temperature ramp tests were performed at 10–40 °C and at a constant strain of 2% and 1 Hz, with a rate of 5 °C/min.
- Time tests: Finally, the hydrogels were evaluated at a temperature of 40 °C during a time interval of 10 min to assess the resistance of the hydrogels at this temperature. These tests were performed with a constant strain of 2% and 1 Hz.

### 2.4. Microstructural Characterization

A Zeiss EVO scanning electron microscope (Zeiss, Jena, Germany) was used to perform the microstructural characterization of the different hydrogels. Nevertheless, due to the high water content of these systems, they need a pretreatment before observing them in the microscope. This pretreatment consisted of fixing the system with glutaraldehyde and osmium, followed by chemical drying based on acetone solutions. After this stabilization of the samples, they were metallized with a thin layer of gold/palladium to improve their conductivity and improve their visualization in the microscope.

### 2.5. Fourier-Transform Infrared Spectroscopy (FTIR) Measurements

FTIR was carried out in a Hyperion Spectrometer (Bruker, Santa Clara, CA, USA) with an attenuated total reflection (ATR) target. These measurements allow knowing the bonds that make up the systems, since they emit at different wavelengths. Thus, this analysis allows evaluating the differences between the systems. The infrared spectra were obtained at 4000–1250 cm^−1^ with an opening of 100 cm^−1^.

### 2.6. In Vitro Cytotoxicity Assays

The cytotoxicity of hydrogels, at different % (*v*/*v*) values, was estimated in vitro using the CyQUANT™ LDH cytotoxicity assay [26]. Several cell lines from commercial suppliers (ATCC^®^, Manassas, VA, USA) were used, Vero E6 (normal monkey kidney epithelial cells), HeLa (human cervical carcinoma epithelial cells), U937 (human leukemia monocytic cells), THP-1 (human leukemia monocytic cells), and Jurkat (human T leukaemia cells). Each cell line was seeded at 10 × 10^4^ cells/well into Nunc flat-bottomed 96-well plates (ThermoFisher Scientific, Waltham, MA, USA) using complete D-10 or R-10 (Dulbecco’s modified Eagle medium (DMEM) or RPMI supplemented with 10% of fetal bovine serum (FBS), and penicillin, streptomycin, and glutamine), incubated at 37 °C in 5% CO_2_, and used the following day (75 to 90% confluence). FBS used in all experiments was heat inactivated (56 °C, 30 min) prior to use to eliminate complement activity. Hydrogels solutions at different concentration (%, *v*/*v*) values were added to each well and the plates were incubated for 48 h at 37 °C in 5% CO_2_. Controls D-10 or R-10 medium alone was used as negative control. 10 µL of 10X Lysis Buffer, and 10 μL of sterile ultrapure water to each set of triplicate wells were added, used as the Maximum LDH Activity and Spontaneous LDH activity, respectively. Later, the medium from each well was collected by centrifugation of the plate and used to test the cytotoxicity of the hydrogel solutions using CyQUANT™ LDH Cytotoxicity Assay Kit according to manufacturer’s suggestion (Invitrogen™ from Thermo Fisher Scientific, Waltham, MA, USA). The cytotoxicity was measured by fluorescence in a CLARIOstar^®^ (BMG LABTECH, Allmendgrün, Ortenberg, Germany). Each hydrogel concentration was measured in triplicate. The cell viability was calculated by using Equation (1):

(1) $$\%\;\mathrm{Cell}\;\mathrm{viablility}=100-\left( [\frac{\mathrm{Compound}-\mathrm{treated}\;\mathrm{LDH}\;\mathrm{activity}-\mathrm{Spontaneous}\;\mathrm{LDH}\;\mathrm{activity}}{\mathrm{Maximum}\;\mathrm{LDH}\;\mathrm{activity}\;-\;\mathrm{Spontaneous}\;\mathrm{LDH}\;\mathrm{activity}}]\times 100\right)$$

It is worth mentioning that cell viability values were also checked by trypan blue method [37] and no significant differences between both methods were observed.

### 2.7. Statistical Analysis

A statistical study was carried out on each of the selected parameters. For this study, a single-factor analysis of variance (ANOVA) was carried out, using three replicates of each measure independently. Then, a series of statistical parameters were calculated, including the mean and the standard deviation. In addition, a means comparison test was performed to detect significant differences (*p* < 0.05).

## 3. Results and Discussion

### 3.1. Rheological Characterization

Table 1 shows the critical strain of the different hydrogels. As can be seen, the CG systems present higher critical strain values than the CH ones. This could be due to the higher structuration of CH hydrogels, which makes them stronger but less deformable. On the other hand, the combination of both biopolymers generates a synergy when the amount of chitosan is up to 50%, with its critical strain exceeding 100%.

These results are surely due to the fact that chitosan grants them a certain structure, while collagen enables the deformation of the chains without breaking. However, when the chitosan is in a higher proportion (25/75), this synergy is lost, becoming rigid, although improving the values obtained by the unitary chitosan system.

**Figure 2:**
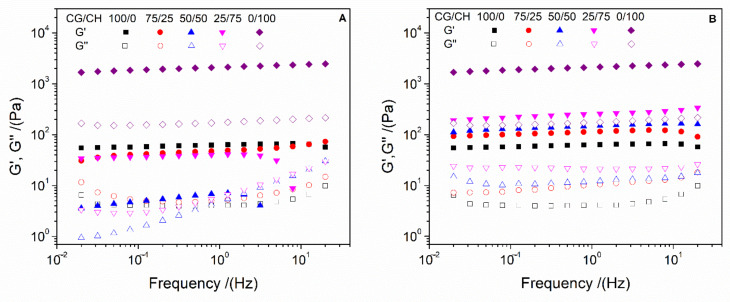
Frequency sweep tests performed at the different systems processed by protocol 1 (**A**) and 2 (**B**).

Figure 2 shows the profile of elastic (G′) and viscous (G″) moduli in the frequency range studied of the different hydrogels. The incorporation of CH to the CG systems by protocol 1 (Figure 2A) seems to harm the stability of the systems. In this way, the incorporation of more than 25% of CH generated the breakdown of the hydrogels at high frequencies, although the unitary CH systems did not break. These results could be due to the fact that the gelation of CH by pH change is slower than that of CG by temperature drop. Thus, a CG-CH network is formed, that is, both biopolymers are in a single network. As the gelation of both biopolymers is different, there is a weakening of the bonds [38]. In addition, increasing the pH in collagen hydrogels decreases the positive charge of the protein chains until repulsive electrostatic interactions become weak enough to allow their stacking and the formation of fibrils [39]. This difference generates areas in the hydrogel where they are not well connected, causing less resistance. Nevertheless, the behavior of the hydrogels processed by protocol 2 (Figure 2B) is different. In this way, the processing of binary hydrogels by protocol 2 confers an intermediate behavior between the unitary systems, being in all cases practically independent of the applied frequency. This difference could be due to the fact that, in this case, CG is completely gelled, forming a three-dimensional network before pH change in which the ungelled CH is in the pores (as it will be seen in the microstructural characterization). By changing the pH, CH gellates thanks to the interconnectivity of the pores [27], producing a network where the pores are joined, improving the resistance of the CG hydrogels.

The differences between the systems can be better evaluated by comparing the elastic modulus and loss tangent at 1 Hz (G′_1_ and tan δ_1_, respectively) which are shown in Table 1. As can be seen, the CH unitary systems presented a G′_1_ much higher than that to the CG ones, although they presented a similar solid character (tan δ_1_ < 1). This difference highlights the better resistance of the CH hydrogels, which can benefit their use as biodegradable biomaterials, although its critical strain is less, which means that CH hydrogels are stiffer than CG ones, giving it a high resistance. However, the great biocompatibility of CG means that it is still the main alternative as long as it has the necessary mechanical resistance. Among the binary systems, processing by protocol 1 reduces G′_1_ value below the values obtained by the CG unitary systems, with the 50/50 system obtaining the lowest values. However, protocol 2 improved the resistance of binary hydrogels, finding intermediate values between the unitary systems, which increased with the amount of CH (25/75 > 50/50 > 75/25). These results are consistent with the hypothesis on their conformation, with the hydrogels processed by protocol 2 being the ones with the best mechanical properties.

**Figure 3:**
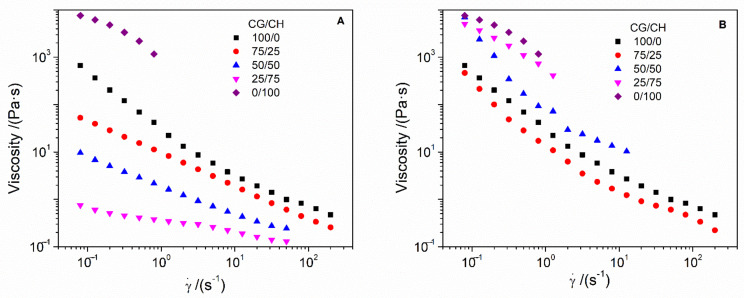
Flow curves obtained by the different systems processed by protocol 1 (**A**) and 2 (**B**).

Figure 3 shows the flow–curve profiles of the different processed hydrogels. All the systems presented a pseudoplastic behavior, where viscosity decreased as the shear rate increases, making the hydrogels more fluid. Regarding the systems, CH was the most viscous, breaking at a shear rate over 1 s^−1^. In Protocol 1 (Figure 3A), the binary systems had lower viscosity values than the unitary systems, such values decreased with the increasing amount of CH in the hydrogels. This may be due to the fact that the physical crosslinking of CG becomes more difficult as more CH is gelling. On the other hand, the binary systems processed by protocol 2 (Figure 3B) have intermediate viscosity values with respect to those of the unitary systems, except for 75/25, which has slightly lower values than the CH systems. In this way, the higher the amount of CH, the higher the viscosity of the systems, causing them to break down at lower shear rates. This higher viscosity is due to a greater strengthening of the CH, which is structured in the pores of the CG network, being greater when there is more CH in the system.

**Figure 4:**
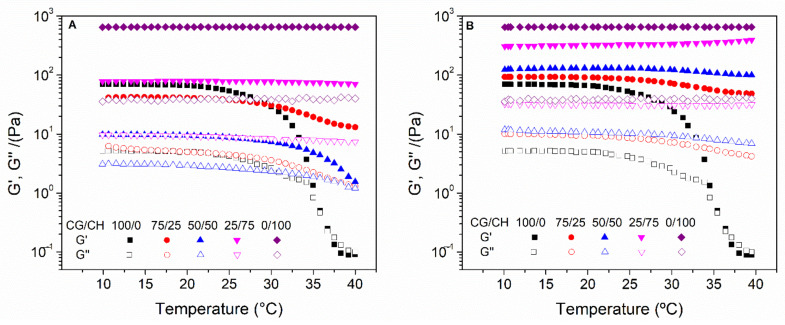
Temperature ramp test carried out by the different systems processed by protocol 1 (**A**) and 2 (**B**).

Figure 4 shows the thermal behavior of the different hydrogels. Firstly, it can be observed that the CH hydrogels are thermally stable, while the CG ones are not. The latter lost their structure from approximately 27 °C. The binary systems showed better thermal stability. Nevertheless, this improvement is greater in protocol 2 than in protocol 1. In this way at least 75% of CH is required to achieve a stable hydrogel at 40 °C by protocol 1, while only 25–50% is necessary in protocol 2. This thermal stability is due to the greater structuring of the systems, thus being more favorable in the systems processed by protocol 2.

**Figure 5:**
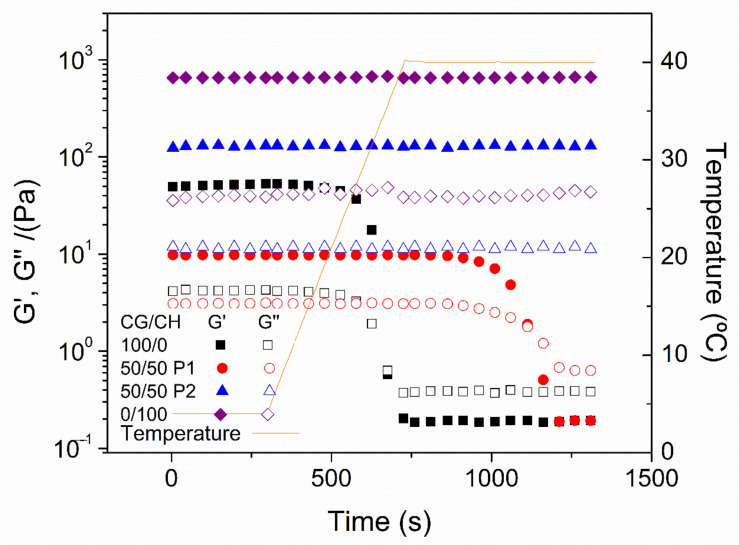
Evaluation of the stability of the selected systems with the temperature.

Thermal stability was further evaluated by maintaining the temperature at 40 °C (Figure 5). As can be seen, the CG unitary systems could not maintain their integrity when the temperature rose above approximately 27 °C. All other systems remained stable at 40 °C. However, the 50/50 system processed by protocol 1 (50/50 P1) did not withstand this temperature for more than 5 min, disintegrated. It can be seen that, although both binary systems have the same CG/CH ratio, the system processed by protocol 2 (50/50 P2) is more stable than that of protocol 1 (50/50 P1). This is due to the greater structuring of the hydrogel of protocol 2, which is consistent with the hypothesis discussed above. This stability was also observed macroscopically (Supplementary Materials, Figure S1), where the CH and 50/50 P1 systems were diluted after 30 min at 40 °C, with the rest remaining constant, although they dried after 24 h at that temperature.

### 3.2. Microstructural Characterization

**Figure 6:**
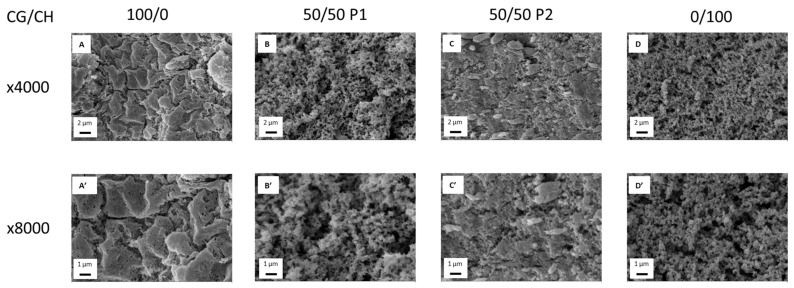
Microstructural images of the selected hydrogels at ×4000 and ×8000: CG/CH 100/0 (**A**,**A′**), CG/CH 50/50 P1 (**B**,**B′**), CG/CH 50/50 P2 (**C**,**C′**) and CG/CH 0/100 (**D**,**D′**).

Figure 6 shows the microstructure of the selected hydrogels. Firstly, there is a great difference between the unitary CG hydrogels (A, A′) and CH hydrogels (D, D′). The CH hydrogels have a sponge-like structure, with high porosity and pore interconnectivity, while the CG hydrogels have a more closed structure, composed of porous grains. In this case, the porosity is given by the cracks between the grains and the small pores present in them, apparently being lower than in the case of CH and, surely, with less pore interconnectivity. This difference can be also seen comparing other studies. Thus, chitosan hydrogels studied by Duan et al. have a sponge-like structure [40], while collagen hydrogels evaluated by Vulpe et al. have a granular microstructure [41].

As for the binary systems, they have a combined structure largely affected by the biopolymer that gels first. In this case, the binary system of protocol 1 (B, B′) has a more similar structure to CH (D, D′) since it is the first to gel with the change of pH. However, the binary system processed by protocol 2 (C, C′) has a more similar structure to CG (A, A′), the biopolymer that first gelled during its cooling at 4 °C. This microstructure is similar to those obtained by Zheng et al., where the author also evaluated collagen/chitosan hydrogels processed by temperature drop [38]. This change in the structure between the binary systems could explain the different mechanical properties observed by the systems elaborated by both protocols, with protocol 2 seeming more favorable.

### 3.3. FTIR Measurements

**Figure 7:**
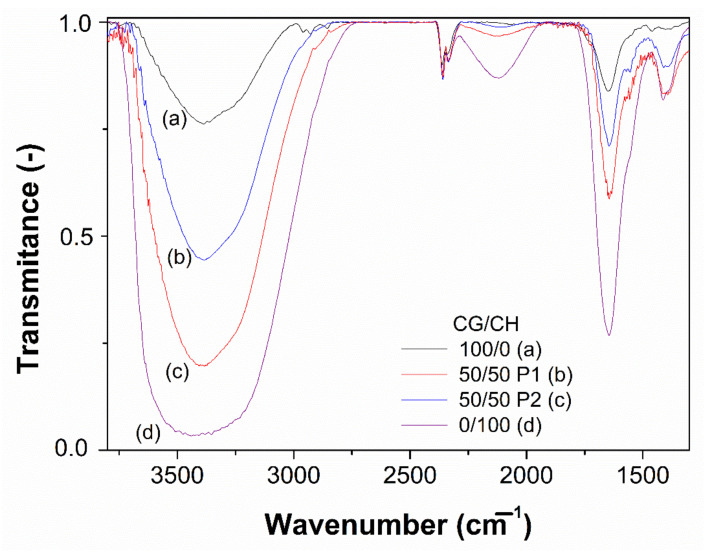
FTIR profile of the selected hydrogels.

Figure 7 shows the FTIR spectra of the selected hydrogels. As can be seen, CG and CH present different profiles due to their different structures. Both systems present a peak between 3750–3100 cm^−1^, which corresponds to N-H and O-H vibrations of the proteins and polysaccharides contain these bonds. However, the CH peak shows a broadening up to 2900 cm^−1^ caused by the presence of C-H groups. In addition, the CH profile has an interesting peak at 2200–2000 cm^−1^, which is characteristic of polysaccharides and does not occur in proteins [42]. This peak is due to the vibrations of C≡C. Finally, both systems, CH and CG, present the peaks of amide I and II (1750–1550 and 1550–1250 cm^−1^, respectively).

The binary hydrogels present an intermediate profile between both unitary systems. Nevertheless, the system processed by protocol 1 (50/50 P1) has a more similar profile to CH, while the one processed by protocol 2 (50/50 P2) is more similar to CG [43]. In this way, this result is consistent with the microstructure observed in the hydrogels and could explain the different behavior presented by the systems processed by the different protocols.

### 3.4. In Vitro Cytotoxicity Assays

The evaluation of the cytotoxicity is essential to develop any kind of material for biomedical applications. The cytotoxicity of the hydrogels investigated was determined by using the LDH assay. 

**Figure 8:**
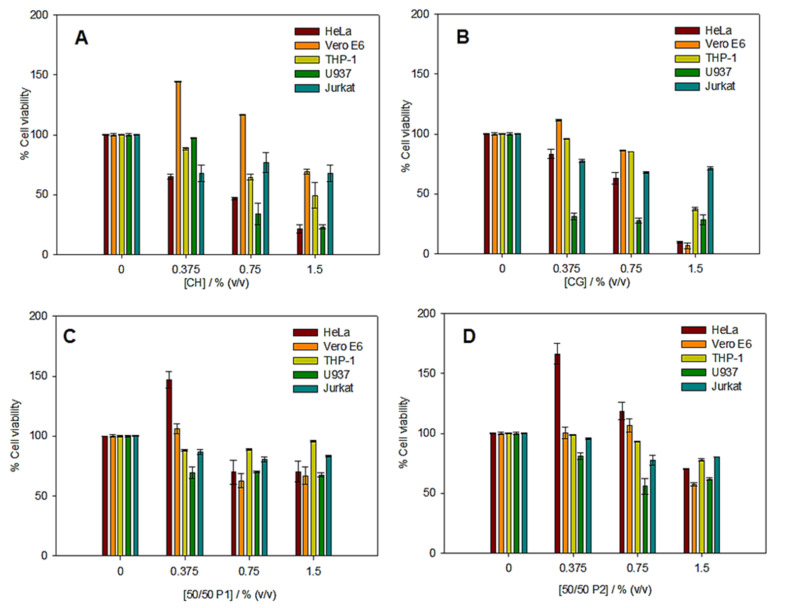
In vitro cytotoxicity results obtained in the selected hydrogels: CG/CH 0/100 (**A**), CG/CH 100/0 (**B**), CG/CH 50/50 P1 (**C**) and CG/CH 50/50 P2 (**D**).

Figure 8 shows the results obtained for each of the systems studied at 48 h. Cells usually have a predominantly negative charge on their membranes, which results in high adhesion to positively charged substrates such as chitosan. Nevertheless, the presence of positive surface charge in CH, together with a high degree of deacetylation, provokes an increase in toxicity due to inhibition cell growth [44,45,46]. The cell viability decreases at higher CH hydrogel concentration as shown in Figure 8A. In this sense, the results obtained for CH hydrogel are in agreement to the above statement. A similar behavior was found for collagen hydrogel (Figure 8B). This trend was also observed by Najafi et al. [46], where the addition of higher collagen concentration reduced the cell growth and increased the in vitro toxicity. On the contrary, CG/CH hydrogels showed a high cell viability (Figure 8C–D). The incorporation of chitosan to collagen hydrogels improves cell viability because CH is resistant to chemical (enzymatic) changes. A prior study has reported that fibroblast cells can grow on CG/CH blended films [47]. This phenomenon has been also observed in the systems investigated (Figure 8C–D). Similar results were obtained for CG/CH P1 and CG/CH P2 hydrogels. Therefore, the toxicity of these systems is not affected by processing of hydrogels and their microstructural and mechanical properties. Thus, the hydrogels formulated are potential candidates for biomedical applications, including tissue engineering and drug delivery.

## 4. Conclusions

The combination of collagen and chitosan in binary hydrogels improved the characteristics of unitary systems. In this way, systems with the thermal stability of chitosan were achieved without compromising the biocompatibility of collagen at a neutral pH, which facilitates their integration as scaffolds for Regenerative Medicine.

Firstly, the importance of the protocol used to develop hydrogels has been highlight. In this way, a pH change followed by a temperature drop (Protocol 1) led to hydrogels with lower G′ values and higher tan δ ones (lower mechanical resistance) than CG system. On the other hand, a temperature drop followed by a pH change (Protocol 2) led to hydrogels with sustainable mechanical resistance and thermal stability. In addition, the microstructure and interactions of these hydrogels also depend on the biopolymer that gels before, favoring its structure. Among the cytotoxicity assays, the change in the protocol do not produce differences in the in vitro biological response of these systems.

Regarding the different systems, collagen system presents a good biocompatibility, although low mechanical resistance. On the other hand, chitosan system has the inverse properties. The different mixing protocols do not present significant differences in the biocompatibility presented, although their mechanical and thermal resistance are highly dependent, enhancing (protocol 2) or worsening (protocol 1) them. Therefore, protocol 2 is the one that achieve the best mechanical properties and thermal resistance, causing synergies between collagen and chitosan since their biocompatibility and mechanical resistance, respectively, are reflected. For this, protocol 2 is the most favorable for regenerative applications.

However, it was a preliminary study of the analysis of the processing stage of the hydrogels, so further physicochemical, mechanical and biological evaluations will be carried out in future studies to evaluate their potential application as scaffolds in Regenerative Medicine.

## Figures and Tables

**Table 1:** Mechanical parameters obtained for the different processed systems.

Protocol	CG/CH Ratio	Critical Strain (%)	G′_1_ (Pa)	tan δ_1_ (-)
1	100/0	63.5 ^a^	63 ^A^	0.065 ^I^
	75/25	>100 ^b^	49 ^AC^	0.107 ^II^
	50/50	>100 ^b^	7 ^B^	0.778 ^III^
	25/75	15.9 ^c^	40 ^C^	0.167 ^IV^
	0/100	10.0 ^d^	2140 ^D^	0.082 ^I, I^
2	100/0	63.5 ^a^	63 ^A^	0.065 ^I^
	75/25	>100 ^b^	115 ^E^	0.092 ^II^
	50/50	>100 ^b^	150 ^F^	0.082 ^I, II^
	25/75	25.3 ^e^	258 ^G^	0.082 ^I, I^
	0/100	10.0 ^d^	2140 ^D^	0.082 ^I, I^

Different symbols included as superscripts in the same column mean significant differences between the systems (*p* < 0.05).

## Data Availability

The data presented in this study are available on request from the corresponding author.

## Supplementary Materials

The following are available online at https://www.mdpi.com/article/10.3390/polym14020272/s1, Figure S1: Macroscopical images of the selected hydrogels subjected at 40 °C with the time.

## References

[1] Hoffman A.S. (2012). Hydrogels for biomedical applications. Adv. Drug Deliv. Rev..

[2] Peppas N.A., Khare A.R. (1993). Preparation, structure and diffusional behavior of hydrogels in controlled release. Adv. Drug Deliv. Rev..

[3] Noferini D., Faraone A., Rossi M., Mamontov E., Fratini E., Baglioni P. (2019). Disentangling Polymer Network and Hydration Water Dynamics in Polyhydroxyethyl Methacrylate Physical and Chemical Hydrogels. J. Phys. Chem. C.

[4] Soto D., Oliva H. (2012). Métodos para preparar hidrogeles químicos y físicos basados en almidón: Una revisión. Rev. Latinoam. Metal. Y Mater..

[5] Choi J.H., Choi O.K., Lee J., Noh J., Lee S., Park A., Rim M.A., Reis R.L., Khang G. (2019). Evaluation of double network hydrogel of poloxamer-heparin/gellan gum for bone marrow stem cells delivery carrier. Colloids Surf. B Biointerfaces.

[6] Rubio-Valle J.F., Perez-Puyana V., Jiménez-Rosado M., Guerrero A., Romero A. (2021). Evaluation of smart gelatin matrices for the development of scaffolds via 3D bioprinting. J. Mech. Behav. Biomed. Mater..

[7] Perez-Puyana V., Jiménez-Rosado M., Romero A., Guerrero A. (2020). Fabrication and characterization of hydrogels based on gelatinised collagen with potential application in tissue engineering. Polymers.

[8] Gull N., Khan S.M., Zahid Butt M.T., Khalid S., Shafiq M., Islam A., Asim S., Hafeez S., Khan R.U. (2019). In vitro study of chitosan-based multi-responsive hydrogels as drug release vehicles: A preclinical study. RSC Adv..

[9] Gull N., Khan S.M., Khalid S., Zia S., Islam A., Sabir A., Sultan M., Hussain F., Khan R.U., Butt M.T.Z. (2020). Designing of biocompatible and biodegradable chitosan based crosslinked hydrogel for in vitro release of encapsulated povidone-iodine: A clinical translation. Int. J. Biol. Macromol..

[10] Milojević M., Harih G., Vihar B., Vajda J., Gradišnik L., Zidarič T., Stana Kleinschek K., Maver U., Maver T. (2021). Hybrid 3D Printing of Advanced Hydrogel-Based Wound Dressings with Tailorable Properties. Pharmaceutics.

[11] Bashari A., Rouhani Shirvan A., Shakeri M. (2018). Cellulose-based hydrogels for personal care products. Polym. Adv. Technol..

[12] Akbari E., Imani R., Shokrollahi P., Heidari keshel S. (2021). Preparation of Nanoparticle-Containing Ring-Implanted Poly(Vinyl Alcohol) Contact Lens for Sustained Release of Hyaluronic Acid. Macromol. Biosci..

[13] Zohreband Z., Adeli M., Zebardasti A. (2021). Self-healable and flexible supramolecular gelatin/MoS2 hydrogels with molecular recognition properties. Int. J. Biol. Macromol..

[14] Zhang Y., Liu M., Pei R. (2021). An in situ Gelling BMSC-laden Collagen/Silk Fibroin Double Network Hydrogel for Cartilage Regeneration. Mater. Adv..

[15] Chopra H., Singh I., Kumar S., Bhattacharya T., Rahman M.H., Akter R., Kabir M.T. (2021). Comprehensive Review on Hydrogels. Curr. Drug Deliv..

[16] Mozafari M., Sefat F., Atala A. (2019). Handbook of Tissue Engineering Scaffolds: Volume One.

[17] Gorgieva S., Kokol V. (2011). Collagen- vs. Gelatine-Based Biomaterials and Their Biocompatibility: Review and Perspectives. Biomaterials Applications for Nanomedicine.

[18] Palmese L.L., Thapa R.K., Sullivan M.O., Kiick K.L. (2019). Hybrid hydrogels for biomedical applications. Curr. Opin. Chem. Eng..

[19] Valencia-Gómez L.E., Martel-Estrada S.A., Vargas-Requena C.L., Rodriguez-González C.A., Olivas-Arnendariz I. (2016). Natural polymers aposites for skin regeneration. Rev. Mex. Ing. Bioméd..

[20] Kanungo I., Fathima N.N., Rao J.R., Nair B.U. (2013). Influence of PCL on the material properties of collagen based biocomposites and in vitro evaluation of drug release. Mater. Sci. Eng. C.

[21] Coombes A.G., Verderio E., Shaw B., Li X., Griffin M., Downes S. (2002). Biocomposites of non-crosslinked natural and synthetic polymers. Biomaterials.

[22] Lv Q., Hu K., Feng Q., Cui F. (2008). Fibroin/collagen hybrid hydrogels with crosslinking method: Preparation, properties, and cytocompatibility. J. Biomed. Mater. Res. Part A.

[23] Perez-Puyana V., Rubio-Valle J.F., Jiménez-Rosado M., Guerrero A., Romero A. (2020). Alternative processing methods of hybrid porous scaffolds based on gelatin and chitosan. J. Mech. Behav. Biomed. Mater..

[24] Khan A., Alamry K.A. (2021). Recent advances of emerging green chitosan-based biomaterials with potential biomedical applications: A review. Carbohydr. Res..

[25] Gull N., Khan S.M., Butt O.M., Islam A., Shah A., Jabeen S., Khan S.U., Khan A., Khan R.U., Butt M.T.Z. (2020). Inflammation targeted chitosan-based hydrogel for controlled release of diclofenac sodium. Int. J. Biol. Macromol..

[26] Fu J., Yang F., Guo Z. (2018). The chitosan hydrogels: From structure to function. New J. Chem..

[27] Sánchez-Cid P., Jiménez-Rosado M., Alonso-González M., Romero A., Perez-Puyana V. (2021). Applied Rheology as Tool for the Assessment of Chitosan Hydrogels for Regenerative Medicine. Polymers.

[28] Franco M.K.K.D., Sepulveda A.F., Vigato A.A., Oshiro A., Machado I.P., Kent B., Clemens D., Yokaichiya F., Araujo D.R. (2020). Supramolecular Structure of Temperature-Dependent Polymeric Hydrogels Modulated by Drug Incorporation. Chem. Sel..

[29] Tan W., Krishnaraj R., Desai T.A. (2001). Evaluation of Nanostructured Composite Collagen–Chitosan Matrices for Tissue Engineering. Tissue Eng..

[30] Deng C., Zhang P., Vulesevic B., Kuraitis D., Li F., Yang A.F., Griffith M., Ruel M., Suuronen E.J. (2010). A Collagen–Chitosan Hydrogel for Endothelial Differentiation and Angiogenesis. Tissue Eng. Part A.

[31] McBane J.E., Vulesevic B., Padavan D.T., McEwan K.A., Korbutt G.S., Suuronen E.J. (2013). Evaluation of a Collagen-Chitosan Hydrogel for Potential Use as a Pro-Angiogenic Site for Islet Transplantation. PLoS ONE.

[32] Gilarska A., Hinz A., Bzowska M., Dyduch G., Kamiński K., Nowakowska M., Lewandowska-Łańcucka J. (2021). Addressing the Osteoporosis Problem—Multifunctional Injectable Hybrid Materials for Controlling Local Bone Tissue Remodeling. ACS Appl. Mater. Interfaces.

[33] Nilsen-Nygaard J., Strand S., Vårum K., Draget K., Nordgård C. (2015). Chitosan: Gels and Interfacial Properties. Polymers.

[34] Jones C.A.R., Liang L., Lin D., Jiao Y., Sun B. (2014). The spatial-temporal characteristics of type I collagen-based extracellular matrix. Soft Matter.

[35] Rebers L., Granse T., Tovar G., Southan A., Borchers K. (2019). Physical Interactions Strengthen Chemical Gelatin Methacryloyl Gels. Gels.

[36] Strober W. (2015). Trypan Blue Exclusion Test of Cell Viability. Curr. Protoc. Immunol..

[37] Zheng T., Tang P., Shen L., Bu H., Li G. (2021). Rheological behavior of collagen/chitosan blended solutions. J. Appl. Polym. Sci..

[38] Coradin T., Wang K., Law T., Trichet L. (2020). Type I Collagen-Fibrin Mixed Hydrogels: Preparation, Properties and Biomedical Applications. Gels.

[39] Duan J., Liang X., Cao Y., Wang S., Zhang L. (2015). High Strength Chitosan Hydrogels with Biocompatibility via New Avenue Based on Constructing Nanofibrous Architecture. Macromolecules.

[40] Vulpe R., Le Cerf D., Dulong V., Popa M., Peptu C., Verestiuc L., Picton L. (2016). Rheological study of in-situ crosslinkable hydrogels based on hyaluronanic acid, collagen and sericin. Mater. Sci. Eng. C.

[41] Riaz T., Zeeshan R., Zarif F., Ilyas K., Muhammad N., Safi S.Z., Rahim A., Rizvi S.A.A., Rehman I.U. (2018). FTIR analysis of natural and synthetic collagen. Appl. Spectrosc. Rev..

[42] Liu L., Wen H., Rao Z., Zhu C., Liu M., Min L., Fan L., Tao S. (2018). Preparation and characterization of chitosan—Collagen peptide/oxidized konjac glucomannan hydrogel. Int. J. Biol. Macromol..

[43] Kean T., Thanou M. (2010). Biodegradation, biodistribution and toxicity of chitosan. Adv. Drug Deliv. Rev..

[44] Schipper N.G.M., Vårum K.M., Stenberg P., Ocklind G., Lennernäs H., Artursson P. (1999). Chitosans as absorption enhancers of poorly absorbable drugs. Eur. J. Pharm. Sci..

[45] Schipper N.G., Varum K.M., Artursson P. (1996). Chitosans as absorption enhancers for poorly absorbable drugs. 1: Influence of molecular weight and degree of acetylation on drug transport across human intestinal epithelial (Caco-2) cells. Pharm. Res..

[46] Najafi M.F., Vahedi F., Ahmadi S., Madani R., Mehrvarz M. (2008). Effect of Collagen Type I (Rat Tail) on Cell Proliferation and Adhesion of BHK-21. Proceedings of the 4th Kuala Lumpur International Conference on Biomedical Engineering 2008.

[47] Koyano T., Minoura N., Nagura M., Kobayashi K. (1998). Attachment and growth of cultured fibroblast cells on PVA/chitosan-blended hydrogels. J. Biomed. Mater. Res..

