# Supplementary material for: Biocompatible and Thermoresistant Hydrogels Based on Collagen and Chitosan
Source: Polymers (Basel). 2022 Jan 10;14(2):272. doi: 10.3390/polym14020272 (PMC8781623; doi:10.3390/polym14020272)
Supplement: Supplementary file 1 [file polymers-14-00272-s001.zip › polymers-1507840-supplementary-done.pdf]

polymers

MDPI

# Supplementary Material

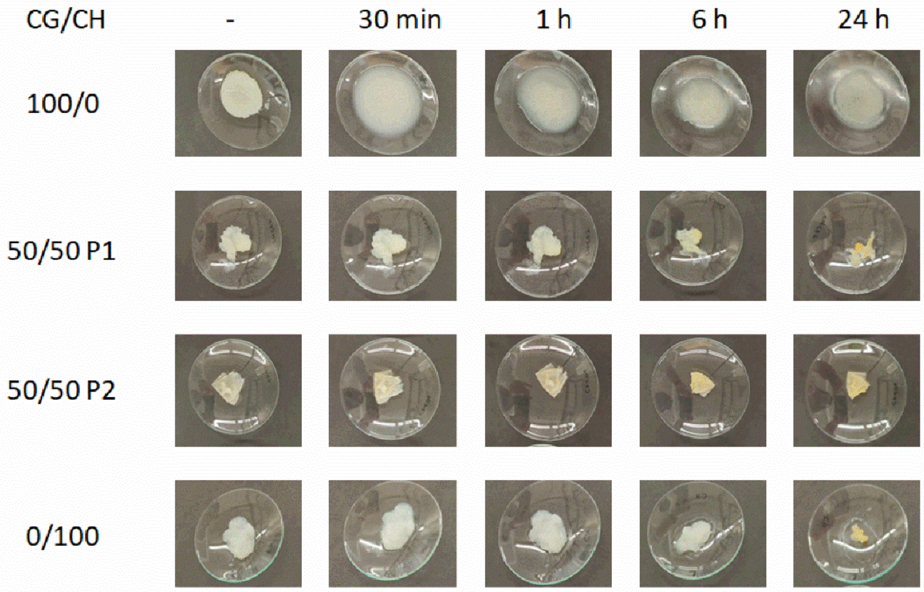


**Figure S1:** Macroscopical images of the selected hydrogels subjected at 40 °C with the time.
